# Reduced Cortisol and Metabolic Responses of Thin Ewes to an Acute Cold Challenge in Mid-Pregnancy: Implications for Animal Physiology and Welfare

**DOI:** 10.1371/journal.pone.0037315

**Published:** 2012-05-25

**Authors:** Else Verbeek, Mark Hope Oliver, Joseph Rupert Waas, Lance Maxwell McLeay, Dominique Blache, Lindsay Ross Matthews

**Affiliations:** 1 Department of Biological Sciences, University of Waikato, Hamilton, New Zealand; 2 Animal Behaviour and Welfare, AgResearch Limited, Hamilton, New Zealand; 3 The Liggins Institute, University of Auckland, Auckland, New Zealand; 4 School of Animal Biology, University of Western Australia, Crawley, Western Australia, Australia; The University of Manchester, United Kingdom

## Abstract

**Background:**

Low food availability leading to reductions in Body Condition Score (BCS; 0 indicates emaciation and 5 obesity) in sheep often coincides with low temperatures associated with the onset of winter in New Zealand. The ability to adapt to reductions in environmental temperature may be impaired in animals with low BCS, in particular during pregnancy when metabolic demand is higher. Here we assess whether BCS affects a pregnant animal's ability to cope with cold challenges.

**Methods:**

Eighteen pregnant ewes with a BCS of 2.7±0.1 were fed to attain low (LBC: BCS2.3±0.1), medium (MBC: BCS3.2±0.2) or high BCS (HBC: BCS3.6±0.2). Shorn ewes were exposed to a 6-h acute cold challenge in a climate-controlled room (wet and windy conditions, 4.4±0.1°C) in mid-pregnancy. Blood samples were collected during the BCS change phase, acute cold challenge and recovery phase.

**Results:**

During the BCS change phase, plasma glucose and leptin concentrations declined while free fatty acids (FFA) increased in LBC compared to MBC (P<0.01, P<0.01 and P<0.05, respectively) and HBC ewes (P<0.05, P<0.01 and P<0.01, respectively). During the cold challenge, plasma cortisol concentrations were lower in LBC than MBC (P<0.05) and HBC ewes (P<0.05), and FFA and insulin concentrations were lower in LBC than HBC ewes (P<0.05 and P<0.001, respectively). Leptin concentrations declined in MBC and HBC ewes while remaining unchanged in LBC ewes (P<0.01). Glucose concentrations and internal body temperature (T_core_) increased in all treatments, although peak T_core_ tended to be higher in HBC ewes (P<0.1). During the recovery phase, T4 concentrations were lower in LBC ewes (P<0.05).

**Conclusion:**

Even though all ewes were able to increase T_core_ and mobilize glucose, low BCS animals had considerably reduced cortisol and metabolic responses to a cold challenge in mid-pregnancy, suggesting that their ability to adapt to cold challenges through some of the expected pathways was reduced.

## Introduction

Grazing sheep in temperate regions such as New Zealand can face a number of environmental challenges simultaneously. Common challenges met by pregnant sheep are long-term undernutrition leading to a loss of body reserves, due to poor pasture quality and growth during the winter months and lack of adequate nutritional supplementation. Such winter undernutrition periods often occur simultaneously with low temperatures, high rainfall and strong winds. Pregnancy in sheep normally occurs in winter and is therefore likely to coincide with periods of low food availability and low temperatures. Pregnancy is a metabolically demanding physiological state and could increase the animals' vulnerability to environmental challenges. In addition, certain agricultural practices may further exacerbate the environmental burden. For example, it is common practice in New Zealand to shear ewes in mid-pregnancy (which coincides with mid-winter) in order to attempt to increase the birth weights of the lambs [1], which could potentially reduce the pregnant sheep's ability to cope with cold challenges. Therefore, low body reserves combined with cold challenges may lead to an increased risk of compromised welfare in pregnant ewes.

Challenges that threaten energy homeostasis, such as cold exposure, will normally lead to activation of the hypothalamic-pituitary-adrenal (HPA) axis resulting in the release of cortisol [2], [3]. Cortisol facilitates the mobilisation of energy substrates and supports energy homeostasis [3]. Therefore, it is important that an appropriate HPA-axis stress response is mounted during metabolically demanding challenges. However, HPA-axis responses to stressors are attenuated during pregnancy [4], [5], which could have implications for pregnant animals exposed to cold challenges.

Animals generally adapt to cold exposure by increasing food intake [6] and resting metabolic rate [6], [7]. However, ewes with low body reserves and limited access to food may have insufficient substrates to conserve or generate heat (thermogenesis). Non-pregnant ewes with low Body Condition Score (BCS, a measure of body reserves) also have lower levels of plasma insulin, leptin, glucose and insulin like growth factor 1 (IGF-1) [8], [9], [10], [11] and higher plasma free fatty acids (FFA) and β-hydroxybutyrate (β-HBA) concentrations compared to ewes with a moderate or high BCS [9]. These metabolic and endocrine characteristics led us to predict that pregnant ewes with low BCS and limited food availability may have a reduced ability to mobilize energy substrates during acute cold challenges compared to ewes with a moderate or high BCS that have sufficient food available.

A healthy and productive animal is characterised by the ability to anticipate and respond to changes in its environment; the welfare of animals is reduced when there is an inability to respond appropriately [12]. However, the impact of different challenges applied simultaneously on animal health and welfare has rarely been investigated. Low BCS and cold exposure are two challenges that are likely to overlap on a relatively regular basis while ewes are pregnant, making them relevant for investigating the impact of simultaneous challenges. However, we do not aim to assess the impacts of pregnancy on stress responses *per se*, but aim to use pregnant ewes because of their high metabolic demands, which may increase their risk of impaired welfare during metabolically demanding challenges. We hypothesise that pregnant ewes with limited energy availability due to low BCS may have a reduced ability to mount HPA-axis (cortisol) and metabolic responses to a cold challenge and consequently may have a reduced ability to mobilize energy substrates, which could compromise their health and welfare. Therefore, we aim to create different BCS groups by feeding pregnant ewes at low, moderate and high levels of intake and to investigate the effects of BCS on stress and metabolic responses to an acute cold challenge.

## Methods

### Ethics statement

This study was approved by the Ruakura Animal Ethics Committee and the University of Waikato Animal Ethics Committee. Animals were closely monitored throughout the experiment and no animal health or welfare issues were observed during or after the experiment. The treatments imposed were designed to detect important differences in the ability to adapt to cold challenges, without compromising long-term animal health and welfare. The cold challenge was designed to simulate winter conditions that are typical for the New Zealand climate, similar to conditions that farmed pregnant sheep encounter on a regular basis while grazing.

### Animals and management

A base flock of 100 (4–5 year old) Coopworth×Texel ewes was initially maintained on pasture and supplemented with a complete pelleted ration (9.8 MJ/kg dry matter containing 65% lucerne and 30% barley with the remainder consisting of limestone, molasses and trace elements; CamTech, Hamilton, New Zealand). Mating was synchronized using Eazi-breed™ CIDRS® (intravaginal Controlled Internal Drug Release Devices containing 0.3 g progesterone, Pfizer Animal Health, Auckland, New Zealand) and ewes were mated in two different groups (50 ewes per group); group 1 was mated in early April 2008 and group 2 three weeks later in late April 2008 (southern hemisphere autumn). Ewes should have been cycling 48 h after CIDR removal [13] and this day was taken as day 1 of pregnancy (normal term is 148–150 days). Rams equipped with harnesses and crayons were present at CIDR removal and allowed to mate for three consecutive days. Pregnancy and the number of fetuses were confirmed on day 41 and 62 of pregnancy by ultrasound scanning. All ewes were kept on pasture from February 2008 (60 days prior to mating, southern hemisphere summer) until June 2008 (day 60 of pregnancy, southern Hemisphere winter) and were then housed in individual pens (1.3 m×1.0 m×1.0 m high constructed of steel posts and mesh with water available *ad libitum*) until the cold challenge (see below). They continued to receive the same pelleted ration as on pasture and an additional 100 g of hay per day. The average environmental temperatures in 2008 were 19.1°C in February, 18.2°C in March, 15.2°C in April, 10.4°C in May and 9.8°C in June [14].

### Experimental design

**Figure 1:**
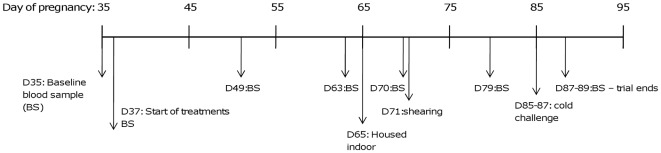
Experimental timeline (D = day of gestation, BS = blood sample).

In order to assess the impact of BCS on the metabolic and stress responses to cold exposure, ewes with an initial BCS of 2.7±0.1 and live weight (LW) of 55±2 kg were divided into three groups at day 37 of pregnancy (first trimester) and fed to attain a low BCS (LBC, aimed at BCS 2), medium BCS (MBC, aimed at BCS 3) or high BCS (HBC, aimed at BCS 4) by day 80 of pregnancy (denoted as the BCS change phase), with six ewes per treatment (see below for BCS scoring criteria). Plasma concentrations of endocrine and metabolic parameters were measured at regular intervals during the BCS change phase. On either day 85 or 87 of pregnancy (second trimester), the ewes were exposed to a 6-h acute cold challenge in a climate-controlled room (4.5°C) equipped with water sprinklers and wind fans (see Figure 1 for the experimental time line). After the challenge, the animals were returned to the indoor housing facility (denoted as the recovery phase). Blood samples were collected at regular intervals during the cold challenge and the recovery phase. All ewes were shorn 2 weeks prior to the acute cold challenge (Figure 1) and the fleeces were collected and weighed.

### Body condition score change phase

Feeding levels were adjusted to alter BCS and LBC, MBC and HBC sheep were offered food at 65%, 130% and 200% of theoretical maintenance requirements, respectively [15], starting at day 37 of pregnancy. Food intake on pasture was controlled by keeping ewes in groups in small paddocks with limited grass availability, so that they were reliant on the pellet supplementation to attain the desired BCS. Feed was provided once a day between 0830 and 1130 h. The daily ration was adjusted weekly depending on BCS gain/loss in order to reach the target BCS. During indoor housing, ewes were fed appropriate amounts of the pelleted ration once a day between 0830 and 1130 h according to individual needs to attain the targeted BCS. Mean food intake was 577±90 g for LBC ewes, 1992±209 g for MBC ewes and ad libitum in HBC ewes (offered food was 2921±273 g) between day 60 and 90 of pregnancy.

BCS was assessed on a 6 point scale according to the system developed by Russell et al. [16]. According to this system, BCS is assessed by manual palpitation of the prominence of the spineous processes of the anterior lumbar vertebrae, the sharpness and degree of cover of the ends of the transverse processes, and the amount of fat and muscle tissue between the transverse and spineous processes. A BCS score of 0 indicates an emaciated animal at the point of death and a BCS score of 5 an extremely obese animal. BCS was scored by the same trained assessor once to twice a week. A second trained assessor who was blind to the treatments scored the ewes independently once every three weeks. The scores between the two assessors were then compared and, in case of any disagreements on the score of a particular animal, the animal was scored again until a final score was agreed upon. There was 80% agreement in independent scores between the two assessors.

Body composition was assessed by ultrasound scanning [17], [18]. The width (A-measure) and depth (B-measure) of the eye muscle (*m. longissimus dorsi*), the thickness of the fat covering the eye muscle (C-measure) and the thickness of the tissue 110 mm lateral to the mid-line over the 12^th^ rib (GR-measure) were measured on day 35, 56 and 76 of pregnancy. Live weight was measured weekly until the end of the study.

Ewes were shorn at day 71 of pregnancy, after they had been habituated to the indoor housing conditions (Figure 1). Therefore, the fleece present during the grazing period would have prevented acclimatization to cold conditions prior to the cold challenge. The environmental temperature in the indoor housing facility was recorded every 10 min during the indoor housing period (25 days) by HOBO Pro Dataloggers (Onset Computer Corporation, Bourne, MA, USA) and the average temperature was 10.8±2.7°C.

A modified CIDR® fitted with a microprocessor-controlled MinilogTX data logger (Vemco Ltd., Shad Bay, Nova Scotia, Canada) was inserted into the vagina prior to the cold challenge. The logger recorded maternal body temperature (T_core_) every 10 min. Blood samples were collected at day 35 (baseline), 37, 49, 72, 79 and 86 or 88 of pregnancy (Figure 1). Food was removed at 1700 h the day before each sample was taken in order to avoid short-term variations in plasma endocrine and metabolite concentrations due to short-term feeding session effects. For periods when the sheep were kept at pasture, the animals were held indoors (in familiar group pens) the night before samples were taken. All blood samples were taken between 0800 and 1100 h, before feeding and were stored on ice immediately after collection. After centrifugation, plasma was stored at −20°C until analysis.

### Acute cold challenge

Six ewes per treatment that were scanned as carrying a single fetus and that had achieved the target BCS or that were closest to the target BCS by day 80 of pregnancy were selected from the base flock. However, only four ewes in the LBC group and four ewes in the MBC group gave birth to single lambs, the remaining two ewes gave birth to twin lambs. All ewes in the HBC group gave birth to single lambs. Ewes were exposed to a 6-h acute cold challenge at day 85 or 87 of pregnancy (Figure 1). Ewes were divided into four subgroups balanced for BCS, and were tested on separate days (between 3 and 5 ewes per testing day). Intracath™ blood sampling devices consisting of 12G needles and 040 PVC tubing (Biocorp, Huntingdale, Australia) were inserted into the jugular vein 2 days before the cold challenge under local anaesthesia. For each ewe, six calibrated I-buttons (Kooltrak Standard Temperature Logger Unmounted, Kooltrak GmbH, Geisenheim, Germany) were attached to electrocardiograph (ECG) patches and glued to 2 to 3 cm^2^ areas of closely clipped skin (one on the base of each ear, one on each side of the mid-trunk and one on each hind leg). The I-buttons recorded skin temperature every 10 min, starting 30 min before and ending 120 min after the challenge.

Before the start of the cold challenge, ewes were moved to an environmental chamber (to which they had been exposed previously) and placed in metabolic crates at ambient temperature (11.8–14.7°C). Sheep were not given any food prior to the cold challenge. A baseline blood sample was taken 1 h after entering the chamber. The acute cold challenge started 2 h after the baseline blood sample (time was set to 0 min at the start of the cold challenge). The temperature of the chamber was set to 4.5°C at −10 min and reached this temperature within 10 min. At 0 min, the mist sprinklers and wind fans were activated and the sheep were wetted with approximately 10 L of water from a standard plastic bucket. Temperature and wind data were recorded with a Kestrel® 3000 pocket wind meter (Nielsen-Kellerman, Boothwyn, USA). The fans produced wind for the full 6 h (average 0.5 m/s, minimum 0.2 m/s and maximum 3.2 m/s) and mist sprinklers were activated every 30 min for 5 min producing 15.4 mm/s of water to keep ewes moist. Blood samples were collected at 1, 10, 20, 30, 40, 50, 60, 90, 120, 150, 180, 240, 300 and 360 min. After the last blood sample had been collected the chamber temperature was increased to 18°C and the ewes were towel dried and moved back to their home pens within 30 min. Recovery blood samples were taken 2 h post-challenge and the following morning around 0900 h (day 86 or 88 of pregnancy). Ewes were fed immediately after collecting the 2 h post-challenge sample.

### Blood sample analysis

Glucose was analysed by enzymatic colorimetric assay (Roche, Mannheim, Germany), urea by kinetic UV assay (Roche), FFA by enzymatic colorimetric assay (Randox Laboratories Ltd, Ardmore, Crumlin, UK) and β-HBA by kinetic UV assay (Randox). The average intra-assay coefficients of variation (CV) were 2.2, 1.7, 2.7, and 3.2% for glucose, urea, free fatty acids, and β-HBA, respectively.

Plasma hormone concentrations were measured by specific radioimmunoassay (RIA). Plasma insulin was measured according to previously published methods [19] except that ovine insulin was used as the standard (Sigma Chemical, St. Louis, MO, batch # I9254). The minimal detectable concentration was 0.03 ng/ml plasma and the inter- and intra-assay CV values were 9.3% and 12.4%, respectively. Plasma IGF-I was measured using an insulin-like growth factor binding protein (IGFBP)-blocked RIA [20], [21]. The detection limit was 0.7 ng/ml and the inter- and intra-assay CV were 9.5% and 10.0%, respectively. Cortisol was measured using mass spectrometry according to previously published methods [22]; mean inter- and intra-assay CV values were 11.2% and 7.1%, respectively.

Plasma leptin concentrations were measured in duplicate by double-antibody RIA method [23]. Bovine recombinant leptin (b/o-leptin) was kindly donated by Dr. Ross L. Tellam (CSIRO Tropical Agriculture, Indooroopilly, Queensland, Australia). The minimum detection limit was 0.05 ng/ml. The intra- and inter-assay CV were 4.2% and 8.3%, respectively. Plasma ghrelin was measured in duplicate by a double-antibody RIA method based on the Linco Total Ghrelin RIA Kit. The method was modified according to the method described by Miller et al. [24]. The minimal detection limit was 25 pg/ml. The intra- and inter-assay CV were 4.2% and 4.4%, respectively.

### Statistical analysis

Data are presented as means ± sem or regression slope (95% confidence intervals, CI). When necessary, data were log-transformed for analysis to provide normal residuals (Shapiro-Wilk test). All statistical analyses were carried out using GenStat 13. Metabolic and endocrine responses to changes in BCS over time were analysed with the Residual Maximum Likelihood (REML) procedure using an order 1 or 2 ante-dependence covariance model as appropriate, with BCS treatment as a fixed effect, ewe as a random effect and the baseline at day 35 of pregnancy as a covariate. Initially, we included the number of fetuses as a covariate in the statistical analysis but dropped this from the final model because it was never significant. Additionally, ANOVAS with a Greenhouse-Geisser correction were performed on individual time points with day 35 as a covariate. BCS, LW, fat and muscle data were analysed by repeated measures analysis. In addition, muscle and fat data at day 76 were analysed by a separate ANOVA with day 35 as a covariate to assess absolute differences in fat and muscle reserves before the start of the acute cold challenge. Correlations between BCS, endocrine, fat and muscle data were calculated using the “correlate” procedure in GenStat 13.

The metabolic and endocrine responses to the cold challenge were measured by repeated measures analysis. However, FFA and cortisol showed biphasic responses that could not be properly reflected in a repeated measures analysis. Therefore, a regression analysis was used instead. The Area Under the Curve (AUC; baseline was the value on the y-axis corresponding to the start of the cold challenge) and the highest individual plasma concentration (peak) were calculated for FFA and cortisol. Due to the biphasic responses of FFA and cortisol, we decided to analyse the 0–60 min period separately from the 60–360 min period. The baseline values taken 2 h before the start of the challenge were used as covariates in both the repeated measures and regression analysis. The changes in core (T_core_) and skin (T_skin_) temperature were analysed by repeated measurement analysis with a Greenhouse-Geisser correction. Peak T_core_ was analysed by linear regression of BCS treatment on peak values. Peak T_core_ was defined as the single highest T_core_ value observed between 0 and 360 min of the cold challenge for each individual animal. Orthogonal contrasts were used for all post-hoc comparisons (Bonferroni corrected).

## Results

### Baselines

**Figure 2:**
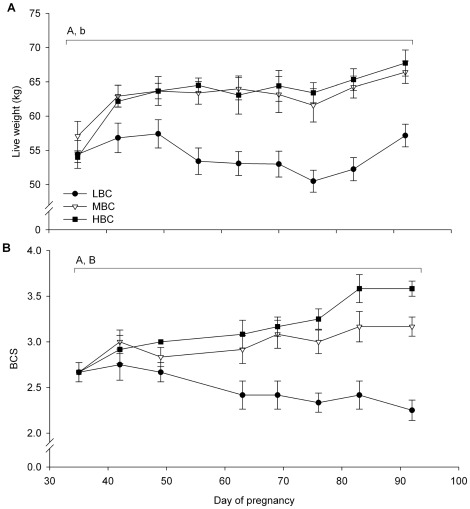
Mean (sem) live weight and BCS for LBC, MBC and HBC ewes between day 37 and 87 of pregnancy: (A) liveweight and (B) BCS. ^A^Effect of BCS treatment (P<0.01). ^B^Time×treatment interaction (P<0.01), ^b^Time×treatment interaction (P<0.05).

**Figure 3:**
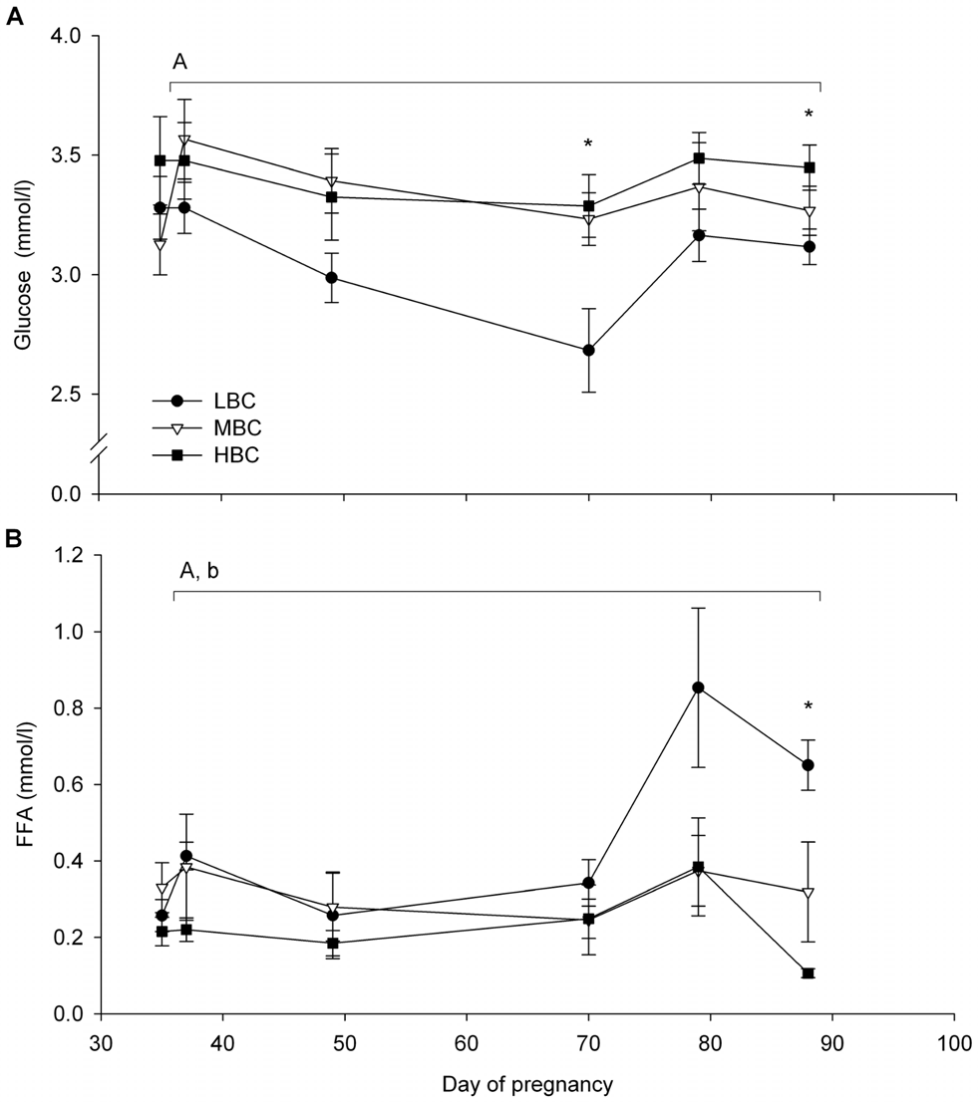
Mean (sem) metabolic responses for LBC, MBC and HBC ewes between day 37 and 87 of pregnancy: (A) glucose and (B) FFA plasma concentrations. ^A^effect of BCS treatment (P<0.01), ^b^Time×BCS treatment interaction (P<0.05). *Indicates a significant BCS treatment difference at individual time points (ANOVA).

**Figure 4:**
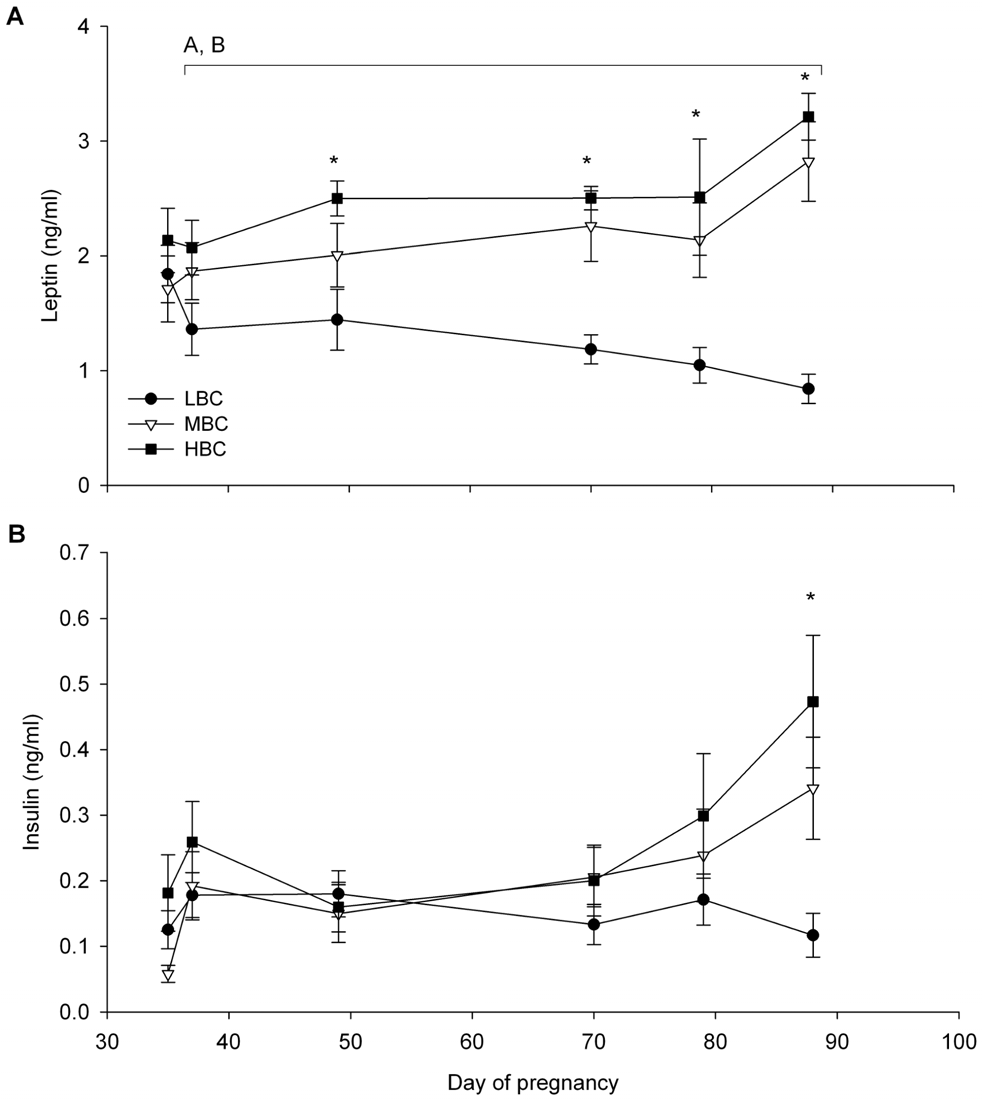
Mean (sem) endocrine responses for LBC, MBC and HBC ewes between day 37 and 87 of pregnancy: (A) leptin and (B) insulin plasma concentrations. ^A^effect of BCS treatment (P<0.01), ^B^Time×BCS treatment interaction (P<0.01). *Indicates a significant BCS treatment difference at individual time points (ANOVA).

LW, BCS (Figure 2), muscle and fat dimensions (Table 1) were not different between BCS groups at day 35 of pregnancy. The plasma concentrations of metabolic and endocrine parameters also did not differ between BCS groups at day 35 of pregnancy (Figure 3 and 4, Supporting Information S1). The fleece weights were 2.8±0.1 kg for LBC, 2.9±0.1 kg for MBC and 2.8±0.2 kg for the HBC ewes, the difference was not significant.

**Table 1:** Mean ± SEM eye muscle width (mm), depth (mm), fat (mm) and GR depth (mm) for LBC, MBC and HBC ewes at day 35 (Baseline), 56 and 76 of pregnancy.

	day	35	56	76	BCS effect	Time effect	Time×treatment
Muscle width	LBC	55±3	54±2	53±2^a^	ns	ns	ns
	MBC	54±1	56±1	57±2a,b			
	HBC	53±0	58±2	57±1^b^			
Muscle depth	LBC	28±2	27±1	27±1^a^	ns	ns	P<0.1
	MBC	27±1	29±1	30±2^b^			
	HBC	26±0	30±1	29±1^b^			
Muscle fat	LBC	6.0±0.7	5.7±0.8	5.0±0.5^A^	ns	ns	P<0.05
	MBC	5.5±0.4	6.3±0.5	6.3±0.7A,B			
	HBC	5.8±0.8	6.5±0.4	8.0±0.7^B^			
GR depth	LBC	11.5±1.1	11.7±1.3	12.5±1.4^A^	ns	P<0.001	P<0.05
	MBC	9.8±0.9	12.7±1.0	16.3±1.5^B^			
	HBC	12.2±0.7	15.0±1.4	17.5±0.6^B^			

a,b Mean values within a column with different superscript letters are significantly different (lower case P<0.05, upper case P<0.01).

### Body condition score change phase

BCS and LW were lower in LBC ewes than MBC (both P<0.001) and HBC ewes (both P<0.001) between day 35 and 83 of pregnancy (Figure 2); there was also a time×BCS treatment interaction for BCS (P<0.001) and LW (P<0.05), with LBC ewes decreasing (BCS) or showing little change (LW) and MBC and HBC ewes increasing BCS and LW. There was no difference in BCS and LW between MBC and HBC ewes.

Eye muscle width (P<0.05), depth (P<0.05), fat cover (P<0.01) and GR depth (P<0.01) were influenced by BCS treatments at day 76 of pregnancy, with LBC ewes having lower muscle and fat reserves compared to MBC and HBC ewes (Table 1). In addition, there were time×treatment interactions for muscle fat and GR depth (both P<0.05), with LBC ewes showing little change and MBC and HBC ewes increasing fat tissue (Table 1).

BCS was correlated to muscle width (r = 0.55, P<0.05), muscle fat (r = 0.78, P<0.01) and GR depth (r = 0.78, P<0.01), and tended to be correlated to muscle depth (r = 0.52, P<0.1) at day 76 of pregnancy.

During the BCS change phase, plasma glucose concentrations were lower in LBC ewes compared to MBC (P<0.01) and HBC ewes (P<0.05, Figure 3A); MBC and HBC ewes were not different. Plasma FFA concentrations were higher in LBC compared to MBC (P<0.05) and HBC ewes (P<0.01), while there was no difference between MBC and HBC ewes (Figure 3B). A time×BCS treatment interaction was found for plasma FFA concentrations (P<0.05) with FFA concentrations increasing in LBC ewes compared to MBC and HBC ewes. Plasma leptin concentrations were lower in LBC ewes compared to MBC ewes (P<0.01) and HBC ewes (P<0.01, Figure 4A); there was no difference between MBC and HBC ewes. There was a time×treatment interaction for plasma leptin concentrations (P<0.01) with LBC ewes showing a reduction and MBC and HBC ewes an increase in leptin concentrations. Plasma insulin concentrations were not affected by BCS between days 35 to 88 days of pregnancy; however, plasma insulin concentrations were lower in LBC ewes at day 88 of pregnancy (P<0.05, Figure 4B). Plasma cortisol, ghrelin, T4, β-HBA and IGF-1 concentrations were not influenced by BCS treatment during the BCS change phase (Supporting Information S1).

BCS was correlated to plasma leptin concentrations (r = 0.83, P<0.001), plasma FFA concentrations (r = −0.65, P<0.01) and plasma insulin concentrations (r = 0.61, P<0.05) and tended to be correlated to plasma glucose concentrations (r = 0.51, P<0.1) at day 88 of pregnancy.

### Acute cold challenge

#### Temperatures

**Figure 5:**
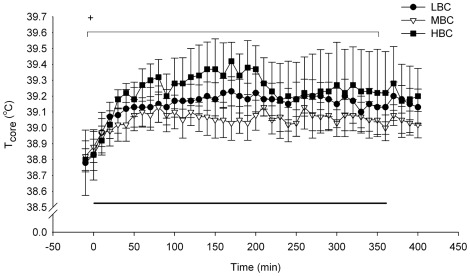
Mean (sem) internal body temperatures (°C) for LBC, MBC and HBC ewes during the acute cold challenge at day 85–87 of pregnancy. The horizontal line indicates the period of the cold challenge (0–360 min). ^+^Tendency for an effect of BCS treatment on peak T_core_ (P<0.1).

**Figure 6:**
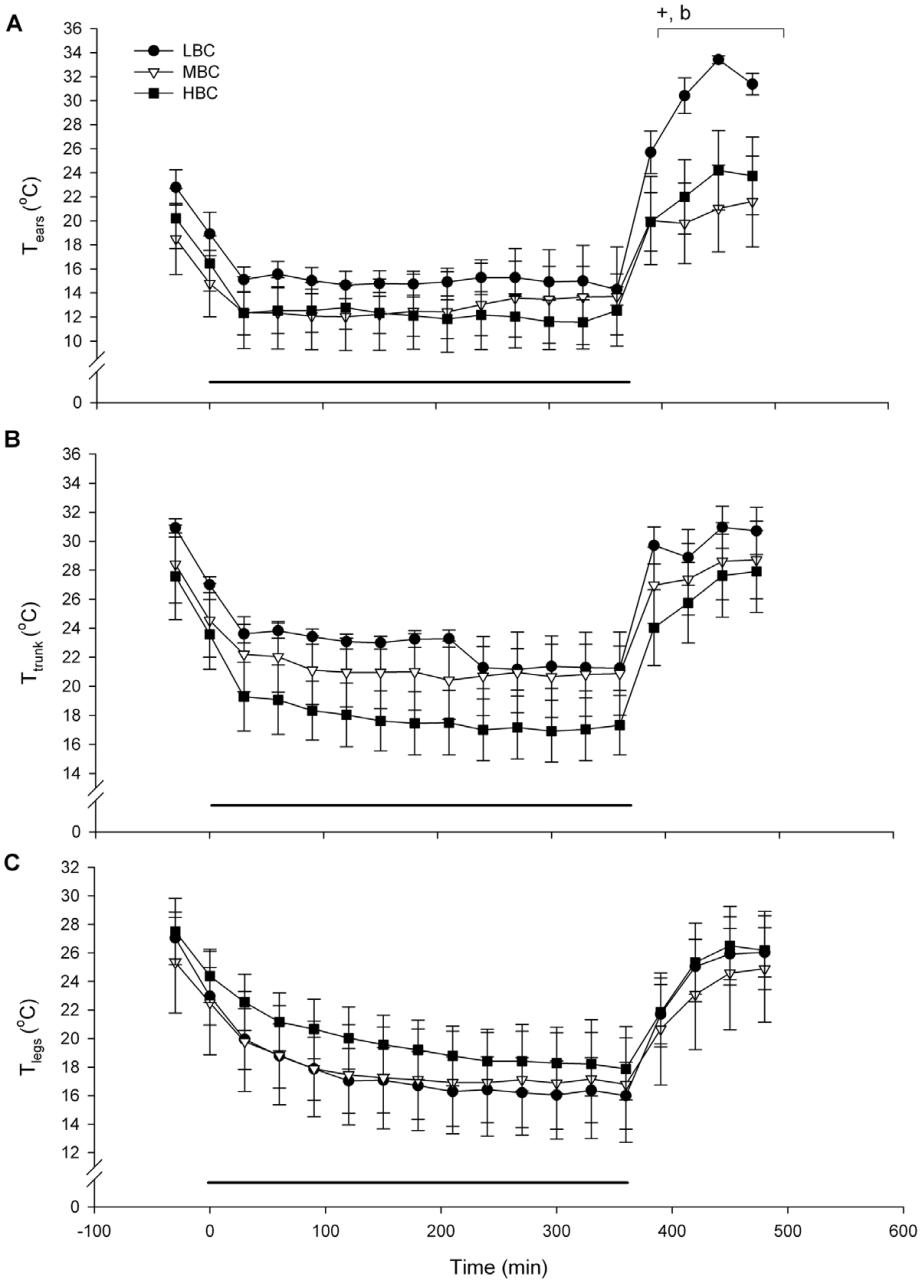
Mean (sem) skin temperatures (°C) during the acute cold challenge for LBC, MBC and HBC ewes at day 85–87 of pregnancy: (A) ear skin temperature, (B) trunk skin temperature and (C) leg skin temperature. The horizontal line indicates the period of the cold challenge (0–360 min). ^+^Tendency for a BCS treatment effect (P<0.1), ^b^Time×BCS treatment interaction (P<0.05).

The average environmental temperature during the acute cold challenge was 4.4±0.1°C. T_core_ of all ewes increased during the acute cold challenge (P<0.001, Figure 5); there was no time×BCS treatment interaction. There was a tendency for a BCS treatment effect on the peak T_core_, with LBC (39.3±0.1°C) and MBC ewes (39.2±0.1°C) tending to have lower peak T_core_ than HBC ewes (39.6±0.2°C, P<0.1); there were no differences in T_core_ after completion of the challenge. T_skin_ was reduced during the acute cold challenge compared to before and after the challenge (P<0.001 for ears, trunk and legs, Figure 6). There was a time×treatment interaction for ear T_skin_ during the recovery phase (P<0.05, Figure 6A), due to a large increase in ear T_skin_ in LBC ewes.

#### Metabolic and stress responses

**Figure 7:**
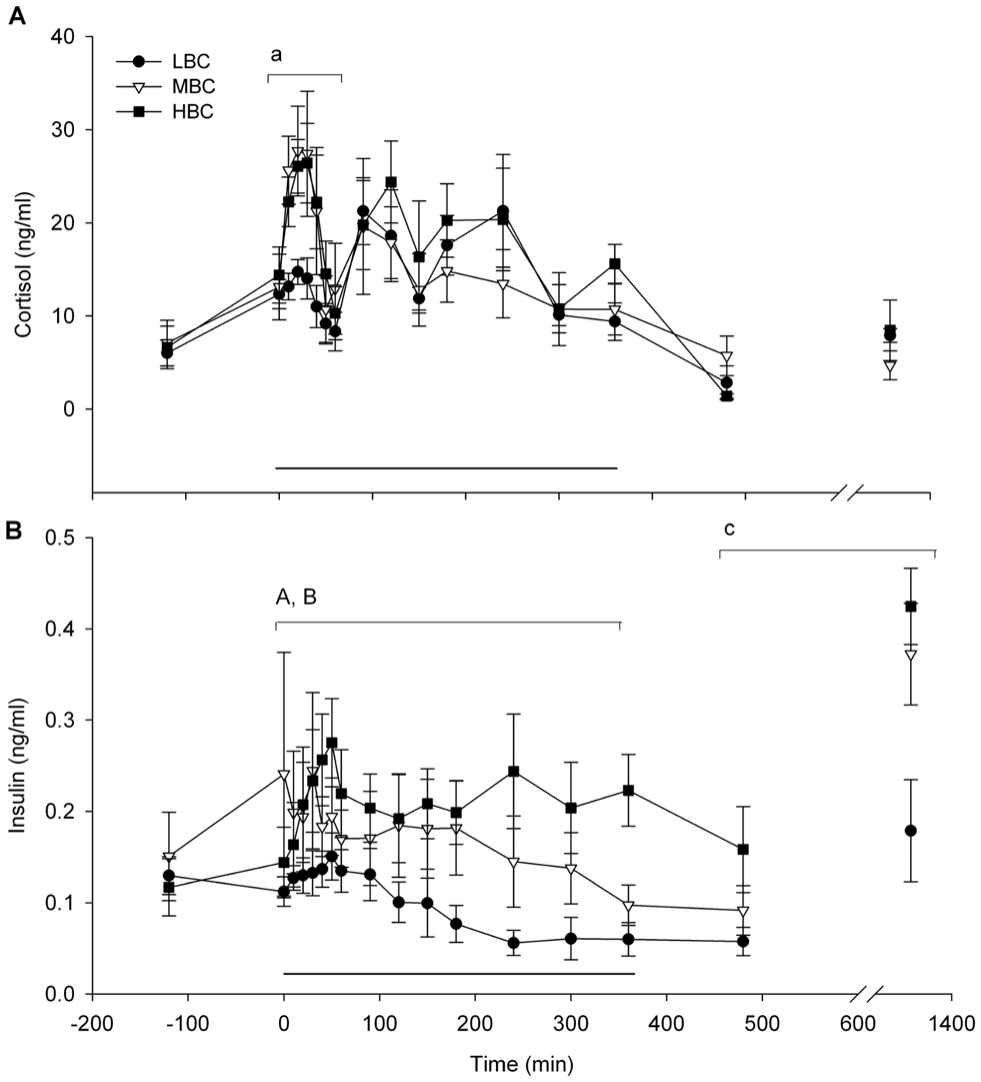
Mean (sem) endocrine responses during the acute cold challenge for LBC, MBC and HBC ewes at day 85–87 of pregnancy: (A) plasma cortisol and (B) insulin responses to the acute cold challenge. The horizontal line indicates the period of the cold challenge (0–360 min). ^a^Effect of BCS treatment on peak plasma cortisol concentrations during the cold stress challenge (P<0.05, see also Table 2), ^A^effect of BCS treatment during the cold stress challenge (P<0.01), ^B^Time×BCS treatment interaction during the cold stress challenge (P<0.01), ^c^Effect of BCS treatment during the recovery phase (P<0.05, 480 and 1320 min).

Plasma cortisol peak height in the first 60 min was less in the LBC ewes compared to MBC and HBC ewes (P<0.05; Figure 7A, Table 2). Area Under the Curve (AUC) and peak plasma cortisol concentrations after 60 min were not different between BCS treatments.

**Table 2:** Regression slope and Confidence interval (CI) for area under the curve (AUC) and peak values, for plasma FFA and cortisol during the acute cold challenge at day 85–87 of pregnancy.

Parameter	AUC		Peak	
	Slope	CI	Slope	CI
*0–60 min*				
FFA (mmol/l)	7.7^a^	6.1	0.0	0.1
Cortisol (ng/ml)	455.2	808.0	14.5^a^	10.83
*60–360 min*				
FFA (mmol/l)	20.7	22.0	0.0	0.1
Cortisol (ng/ml)	−57.19	3832	1.9	13.6

a BCS treatment effect: P<0.05.

Plasma insulin concentrations were greater in HBC than LBC ewes (P<0.001, Figure 7B), and there was a time×treatment interaction with LBC ewes decreasing and HBC ewes increasing plasma insulin concentrations over time (P<0.01).

**Figure 8:**
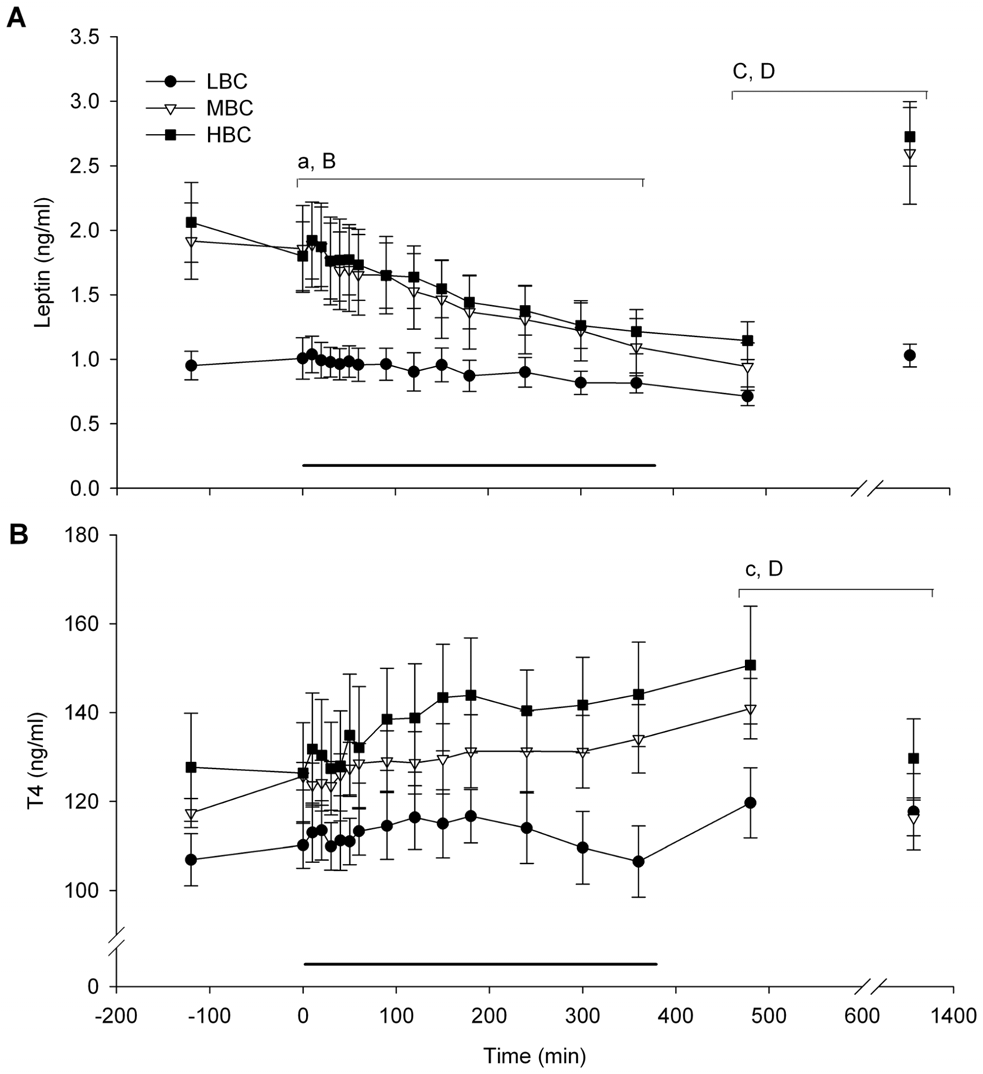
Mean (sem) endocrine responses during the acute cold challenge for LBC, MBC and HBC ewes at day 85–87 of pregnancy: (A) plasma leptin and (B) T4 responses to the acute cold challenge. The horizontal line indicates the period of the cold challenge (0–360 min). ^a^Effect of BCS treatment during the cold stress challenge, (P<0.05), ^B^Time×BCS treatment interaction during the cold stress challenge (P<0.01), ^c^Effect of BCS treatment during the recovery phase (P<0.05, 480 and 1320 min), ^C^Effect of BCS treatment (P<0.01) during the recovery phase, ^D^Time×BCS treatment interaction (P<0.01) during the recovery phase.

Plasma leptin concentrations were less in LBC ewes compared to MBC and HBC ewes (both P<0.05, Figure 8A). In addition, there was a time×treatment interaction for plasma leptin with MBC and HBC ewes decreasing while LBC ewes remained relatively stable over time (P<0.01).

Plasma T4 concentrations were not affected by BCS treatment during the cold challenge (Figure 8B).

**Figure 9:**
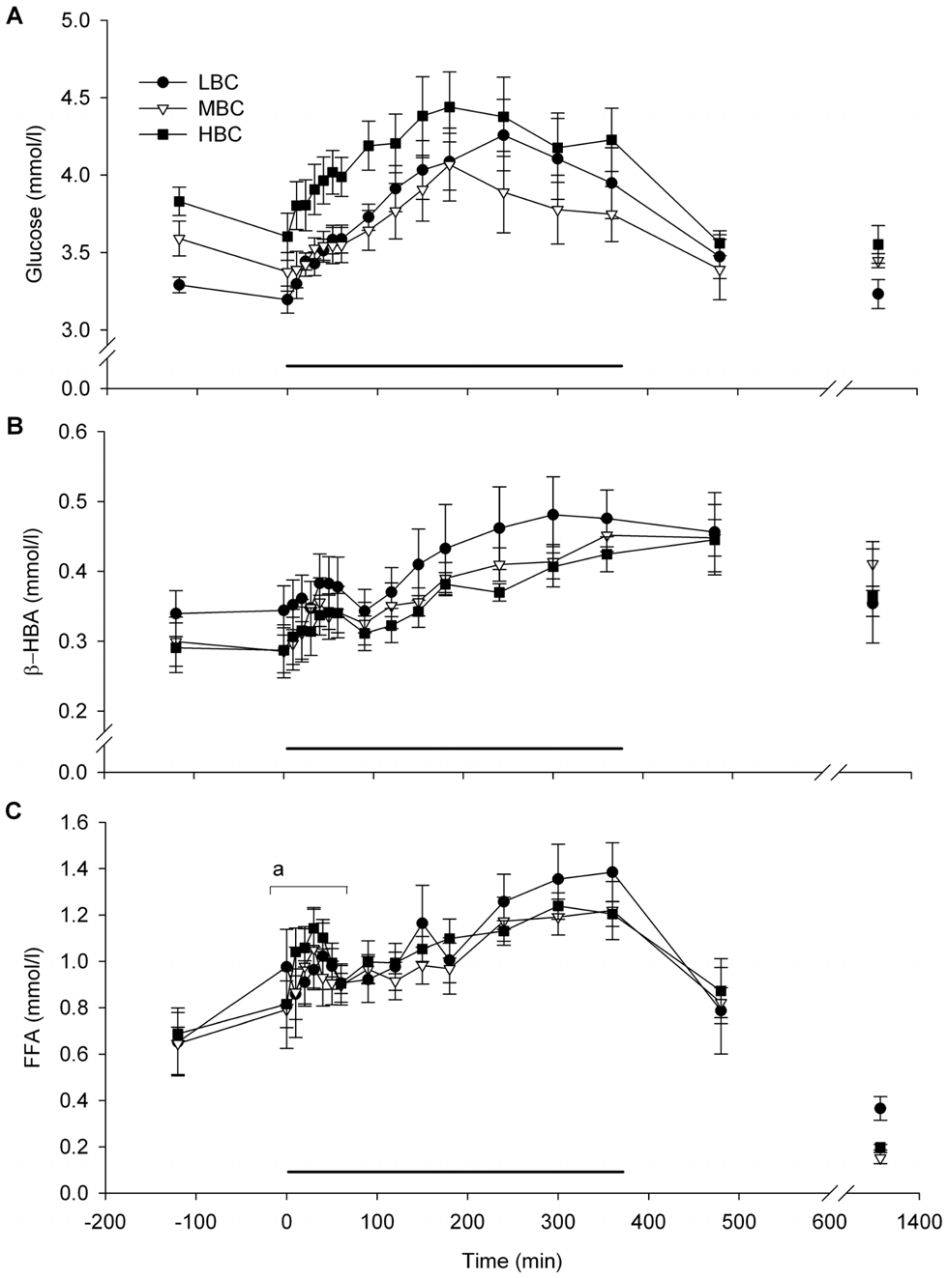
Mean (sem) metabolic responses to the acute cold challenge for LBC, MBC and HBC ewes at day 85–87 of pregnancy: (A) Glucose, (B) β-HBA, and (C) FFA plasma responses to the acute cold challenge. The horizontal line indicates the period of the cold challenge (0–360 min). ^a^Effect of BCS on AUC during the cold stress challenge (P<0.05). For more detailed statistics see Table 2.

The plasma glucose (Figure 9A) and β-HBA (Figure 9B) responses to the acute cold challenge were not affected by BCS treatment. In the first 60 min of the challenge, the AUC of plasma FFA was less in LBC than HBC ewes (P<0.05; Figure 9C, Table 2).

#### Recovery phase

Plasma insulin concentrations increased after the cold challenge (P<0.001) and were less in LBC compared to MBC and HBC ewes (P<0.05, Figure 7B). At 1320 min (22 h), plasma insulin concentrations were higher (0.33 ng/ml) than basal levels measured 2 h prior to the cold challenge (0.13 ng/ml, P<0.001).

Plasma leptin concentrations were less in LBC than in MBC and HBC ewes during the recovery period (P<0.01, Figure 8A), and there was a time×treatment interaction (P<0.01) with leptin concentrations increasing in MBC and HBC ewes but remaining relatively stable in LBC ewes over time. Plasma leptin concentrations were higher at 1320 min (2.11 ng/ml) than at basal levels (1.64 ng/ml, P<0.01)

Plasma T4 concentrations were less in LBC ewes compared to MBC and HBC ewes during the recovery phase (P<0.05, Figure 8B), and a time×treatment interaction (P<0.01) was observed with MBC and HBC ewes decreasing T4 concentrations and LBC ewes showing little change over time. Plasma T4 concentrations at 1320 min (121.3 ng/ml,) tended to be different from basal levels (117.3 ng/ml, P<0.1)

Plasma cortisol, glucose, β-HBA and FFA concentrations were not influenced by BCS during the recovery phase (Figure 7A, 9A, 9B and 9C, respectively).

## Discussion

We combined different feeding levels to alter BCS with an acute cold challenge, and measured the impact on metabolic and stress responses in pregnant sheep. At day 88 of pregnancy, LBC ewes had lower subcutaneous fat depots and muscle size as well as lower plasma glucose, leptin and insulin concentrations and increased FFA concentrations compared to MBC and HBC ewes. The metabolic state of the LBC ewes was therefore consistent with a catabolic state (negative energy balance). During the acute cold challenge, the LBC ewes showed reduced plasma cortisol, FFA, and insulin responses compared to MBC and HBC ewes. Furthermore, plasma leptin concentrations gradually decreased in the MBC and HBC ewes during the cold challenge, while remaining fairly steady in the LBC ewes. However, all ewes were able to mobilize glucose and increase T_core_. During the recovery phase, plasma T4 concentrations gradually declined in MBC and HBC ewes, while little change was observed in the LBC ewes. Overall, the data supported our hypothesis that low BCS ewes on restricted food intake have reduced stress and metabolic responses to an acute cold challenge, suggesting that low BCS ewes have an impaired ability to temporarily adjust their physiology to a challenge. However, the metabolic responses were not sufficiently reduced to compromise T_core_ and energy availability. Therefore, we detected a significant impact of two different environmental challenges applied simultaneously on stress and metabolic responses in pregnant ewes. Future research will have to investigate whether an acute cold challenge will have a similar impact on non-pregnant animals with low BCS. There was very little difference between the MBC and HBC ewes in responses to the cold challenge, most likely because the differences in BCS, LW, body fat and muscle reserves between them were relatively small. The effects of the cold challenge on animal welfare are likely to have been minor, but more severe or repeated cold challenges may aggravate the effects and need further investigation.

### Body condition score change phase

During the BCS change phase, MBC and HBC ewes increased subcutaneous fat depots while LBC ewes did not show any substantial changes. BCS was correlated to measures of body fat and muscle width, suggesting that BCS is associated with body reserves in sheep. It was not possible for us in this experiment to measure other important internal fat depots, such as omental, mesenteric and perirenal fat, which account for a substantial proportion of total body fat in sheep [25], [26]. The lipogenic activity in these tissues varies considerably with perirenal and omental fat having the highest activity and subcutaneous fat having a relatively low activity [27], [28]. Thus, it is likely that LBC ewes had mobilized fat from the omental and perirenal fat depots during BCS loss, which would explain the small change in tissues with lower lipogenic activity such as the GR and eye muscle fat depot. The decrease in plasma leptin and increase in FFA concentrations also suggest that fat depots were being depleted in LBC ewes.

Plasma glucose and leptin concentrations were reduced, and FFA concentrations increased in LBC compared to MBC and HBC ewes, with the differences becoming larger as pregnancy progressed. Plasma insulin concentrations were also reduced in the LBC ewes by day 88 of pregnancy. Several other studies have shown effects of BCS, body fat or feeding level on leptin [29], [30], insulin [31], [32], IGF-1 [33], [34] and cortisol [22], [35] concentrations. Plasma leptin, as well as insulin and FFA concentrations, were also correlated to BCS in this study, which is in agreement with other studies [29], [36]. However, plasma leptin concentrations in MBC and HBC ewes were not as high as reported for pregnant ewes [29] or non-pregnant ewes [36], [37] of similar BCS and feed intake levels; probably because all ewes in our study were fasted 15–18 h prior to blood sample collection to avoid short-term variations in plasma endocrine and metabolite concentrations due to feeding sessions. Plasma cortisol and IGF-1 concentrations were not influenced by BCS in our study. The effects of BCS on metabolic and endocrine parameters depend on the severity of the food restriction regime and the amount of BCS loss [8], [9], [23], [29], [30], so the changes in BCS in this study may have been too small to affect plasma IGF-1 and cortisol concentrations. In addition, there are potential interactions between feed intake level and BCS that may explain the lack of a BCS effect on plasma IGF-1 and cortisol concentrations in the current study. However, all ewes were fasted 15–18 h prior to blood sample collection to avoid short-term variations in plasma endocrine concentrations due to feeding sessions, which should have avoided some of the confounding effects of different feeding levels with BCS.

### Acute cold challenge

The T_core_ increased in response to the cold challenge in all three BCS treatments and remained elevated for the duration of the challenge. Sheep exposed to cold for four days have an increased rectal temperature [38], although decreased rectal temperatures during five weeks of cold exposure have also been reported [6]. Therefore, an increase in T_core_ may be observed as a first response to cold exposure, while T_core_ may decrease after chronic exposure. The HBC ewes showed a tendency for a greater increase in peak T_core_ compared to the other treatments, although the reason for this is uncertain. T_skin_ decreased in all treatments during the cold challenge, but the ear T_skin_ was higher in the LBC ewes than in the MBC and HBC ewes at 2 h post-challenge; LBC ewes could have shivered more (shivering behaviour was not measured), or displayed a higher degree of vasodilatation after completion of the cold challenge leading to increased blood flow and therefore increased T_skin_.

A key novel finding of our study was the significantly smaller plasma cortisol increase within the first 60 min of the cold challenge in the LBC ewes. The activation of the HPA-axis and the release of cortisol in synergy with catecholamines and GH result in increased plasma glucose concentrations and stimulation of lipolysis [3], [39], [40]. Therefore, the increase in plasma cortisol concentrations would have lead to an increase in the availability of energy substrates in all treatments, but to a lesser extent in the LBC ewes. The pattern of cortisol secretion was biphasic in all BCS treatments, with a peak in the first 60 min and a second peak within 120 min. Interestingly, peak cortisol concentrations were reached in the first 60 min in the MBC and HBC ewes, but between 60 and 120 min in the LBC ewes. Stress responses are known to vary across environments and physiological states [2], [41], [42], and the altered metabolic state of ewes with different BCS may have contributed to the difference in the timing and height of the cortisol responses. One explanation for the LBC ewes' reduced cortisol response is to conserve energy during the initial phase of an environmental challenge. Although there is no direct evidence to suggest that the reduced cortisol response in the low BCS ewes is an energy saving mechanism, there is some evidence for the involvement of appetite regulating peptides (e.g., leptin, ghrelin, neuropeptide Y and several others) in HPA-axis modulation [43], suggesting a link between energy availability and stress responses. Therefore, a reduced cortisol response to acute stressors in animals with high metabolic demands and/or limited energy reserves could be an energy saving adaptation.

An alternative explanation for the reduced cortisol response in the LBC ewes is that low BCS is a chronic stressor resulting in permanent changes in the regulation of the HPA-axis. It is well documented that animals under chronic stress do not show elevated basal cortisol levels or even display suppressed basal cortisol levels, but may show enhanced or reduced responses to additional acute stressors [44], [45]. Such alterations in the cortisol response to a challenge is thought to result from HPA-axis down regulation after exposure to repeated or long-lasting stressors [46]. Down regulation of the HPA-axis may avoid the detrimental effects of continuously high cortisol concentrations on key homeostatic processes and/or ensures that sensitivity to novel stressors can still be maintained. In pregnant sheep [35] and lambs [47] it has been shown that the cortisol and adrenocorticotrophic hormone (ACTH) responses, respectively, to a pharmacological stress challenge were reduced after several weeks of food restriction. Therefore, there is evidence that undernutrition results in down-regulation of the HPA-axis in sheep and this could have contributed to the reduced cortisol response in the LBC ewes.

Another particularly interesting finding was the gradual decline of plasma leptin concentrations in the MBC and HBC ewes during the cold challenge, while the LBC ewes' leptin concentrations remained relatively unchanged. In these LBC ewes, this was most likely because levels were already minimal and could not decrease any further. Other studies have also shown a reduction in leptin concentrations during cold exposure in sheep [48] and rats [49], [50]. Our results show that even an acute exposure to cold in animals with sufficient amounts of adipose tissue reduces plasma leptin concentrations to levels that signify the need to increase energy stores. This suggests that hormonal mechanisms responsible for adapting the organism to greater energy needs when ambient temperature drops are sensitive to even relatively short-term temperature changes. This extra-ordinary responsiveness of the leptin system to a thermal challenge is supported by the fact that the recovery of leptin levels begins within several hours after discontinuation of adverse conditions. Therefore, the data show a remarkable example of coupling of the leptin system (as well as - especially in the recovery phase - the insulin system) with the ambient conditions, most likely to facilitate metabolic and behavioural changes needed to cope with the dynamic environment. Importantly, the LBC ewes seemed to be unable to produce a similar hormonal response to the cold challenge, which may be associated with an impaired ability to adapt to low temperatures. This finding has profound consequences for our understanding of BCS and food availability in grazing animals as a welfare aspect of animal husbandry.

The main effect of BCS on plasma T4 concentrations was observed during the recovery phase (largest effect at 2 h after the completion of the cold challenge), and not during the acute cold challenge itself. LBC ewes had lower T4 concentrations and showed little change between 2 h and 14 h post-challenge, while MBC and HBC ewes decreased T4 concentrations. At 14 h post-challenge, there was no longer a difference between BCS treatments and T4 concentrations tended to have returned to baseline levels. These results suggest that T4 responses to a cold challenge may take relatively long to develop and continue after the challenge has been completed. Other studies also report interactions between plasma T4 concentrations, feeding level and the environmental temperature in sheep; food restriction in a warm (23°C) environment for 5 weeks reduced plasma T4 concentrations, while food restriction in a cold environment (0°C) did not change T4 concentrations compared to fully fed sheep [6]. Thyroid hormones may stimulate thermogenesis by altering Na^+^,K^+^ATPase activity resulting in an increased rate of ion transport across the cell membrane [51]. Therefore, the lower plasma T4 concentrations of the LBC ewes as well as the lack of change observed during the recovery phase also suggests that the ability to adapt and recover from a cold challenge may be impaired in LBC ewes.

Acute cold exposure increased plasma insulin concentrations in all ewes, with the HBC ewes showing a significantly larger increase in insulin concentrations compared to LBC ewes. It has been demonstrated that cold exposure reduces insulin secretion compared to a warm environment [52] and increases tissue responsiveness to insulin in sheep [53]. These effects are most likely mediated by the sympathetic nervous-adrenomedullary systems and result in the facilitation of muscle glucose uptake [54]. Therefore, insulin is likely to contribute to the increased availability of energy substrates and the maintenance of energy homeostasis and internal body temperature in animals exposed to cold. The lower increase in plasma insulin concentrations in the LBC ewes could have implications for energy availability and therefore the ability to adapt to reductions in temperature.

### Animal welfare

The response of the HPA-axis during environmental challenges has an adaptive value by facilitating appropriate behavioural reactions [55], [56], [57] and physiological adjustments [3], which are required to maintain or restore energy homeostasis and increase the chances of survival. Therefore, an increase in plasma cortisol concentrations during a stressful challenge may be viewed as successful coping rather than a sign of distress and negative welfare [58]. However, when a stressor is prolonged or when the animal is exposed to several stressors simultaneously, there may be negative consequences for the welfare of animals. The concept of allostasis (i.e., stability through change) may be useful in assessing animal welfare during challenges [59]. Allostasis characterises good animal welfare when an animal responds appropriately to an environmental challenge by (temporarily) adjusting its physiology [12]. However, when the mediators of allostasis (e.g., cortisol) are under- or over-produced or dysregulated, a significant biological cost can be incurred (also called allostatic load), potentially leading to detrimental effects on the animal [60]. Thus, the reduced cortisol response observed in the LBC ewes could be an indication of an increased allostatic load, which could potentially reduce health and welfare. The lower concentrations, or lack of change in concentrations, in endocrine parameters such as insulin, leptin and T4 also suggest that LBC may have a reduced ability to adapt appropriately to cold challenges. However, as stated before, LBC ewes increased T_core_ and mobilized glucose, indicating that they were able to generate heat and mobilize energy substrates for the duration of the challenge. It is likely that allostatic load will increase when LBC ewes are exposed to repeated or long-term challenges, which should be investigated in order to thoroughly assess the impact of low BCS on welfare.

Our experimental design, however, prevented behavioural strategies that animals use to cope with cold (e.g., increasing physical activity, searching for shelter/warm bedding or huddling with conspecifics) because animals were in metabolic crates that restricted physical activity and social contact. Therefore, ewes on farm exposed to cold challenges may be able to use additional behavioural coping strategies that were not captured in the current experiment. It would be important to investigate the extent to which such behavioural strategies are able to buffer some of the physiological impacts of cold exposure observed in the current study. Furthermore, our experimental design was aimed to simulate a situation of chronic undernutrition leading to low BCS that pregnant ewes may experience on farm. Situations in which low BCS ewes are fed at high levels of intake, or when high BCS ewes are fed at very low levels of intake, are less likely to occur on farm and are therefore less relevant from a practical animal welfare perspective. However, investigating the interactions between food intake levels and BCS could potentially provide important information about the biological function of animals simultaneously exposed to food restriction and cold challenges.

### Conclusion

In this study, we assessed the impact of BCS on metabolic and stress responses to an acute cold challenge in pregnant sheep. One of the main findings was the reduced plasma cortisol response in the first 60 min of the acute cold challenge in the low BCS ewes compared to ewes with a moderate or high BCS. The reduced cortisol response is most likely indicative of an energy saving adaptation or of an HPA-axis down regulation. Despite this, LBC ewes were able to mobilize glucose and increase T_core_ during the challenge, indicating that energy homeostasis was maintained. Low BSC ewes also had reduced plasma insulin and leptin concentrations during the cold challenge, as well as reduced concentrations of T4 during the recovery phase. Moreover, LBC ewes showed little change over time in these endocrine parameters in contrast to high BCS ewes. The implications of the reduced metabolic and stress responses to an acute environmental challenge for ewe health and welfare remain to be further investigated, but may indicate that LBC animals have an impaired ability to temporarily adjust their physiology to cold, wet and windy conditions. An impaired ability to adapt to environmental challenges such as cold could increase the risk of impaired welfare in grazing pregnant sheep with low BCS in temperate regions. Further investigations using different types or durations of challenges would be useful in assessing the impact of several simultaneous challenges on animal health and welfare.

## Supporting Information

Supporting Information S1 Mean (sem) endocrine responses for LBC (rhombus), MBC (square) and HBC (triangle) ewes between day 37 and 87 of pregnancy: (A) Cortisol, (B) Ghrelin and (C) T4 (D) β-HBA and (E) IGF-1 plasma concentrations.
(TIF)
