# Supplementary figures and images for: Reduced Cortisol and Metabolic Responses of Thin Ewes to an Acute Cold Challenge in Mid-Pregnancy: Implications for Animal Physiology and Welfare
Source: PLoS One. 2012 May 25;7(5):e37315. doi: 10.1371/journal.pone.0037315 (PMC3360704; doi:10.1371/journal.pone.0037315)

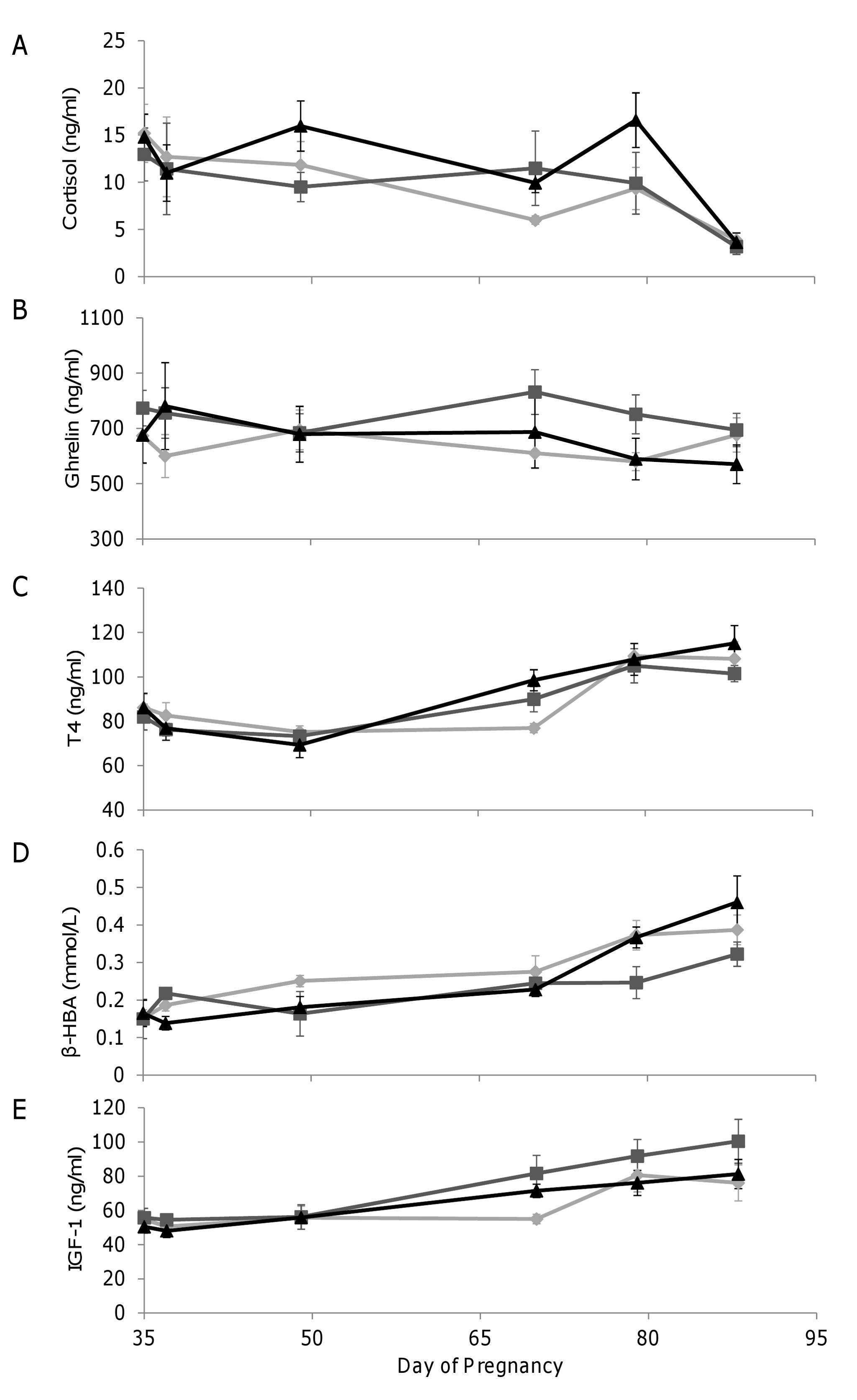

Supplement: Supporting Information S1 — Mean (sem) endocrine responses for LBC (rhombus), MBC (square) and HBC (triangle) ewes between day 37 and 87 of pregnancy: (A) Cortisol, (B) Ghrelin and (C) T4 (D) β-HBA and (E) IGF-1 plasma concentrations. (TIF) [file pone.0037315.s001.tif]
